# Combined omalizumab and desensitization to control IgE-mediated hypersensitivity in enzyme replacement therapy for late-onset Pompe disease

**DOI:** 10.1186/s13023-026-04385-4

**Published:** 2026-05-14

**Authors:** Alberto Lerario, Elena Abati, Monica Sciacco, Giacomo Pietro Comi, Vanessa Desantis, Simona D’Amore, Antonio Giovanni Solimando, Roberto Ria, Stefania Corti, Federico Spataro

**Affiliations:** 1 Neuromuscular and Rare Disease Unit, Fondazione IRCCS Ca’ Granda Ospedale Maggiore Policlinico, 20122 Milano, Italy; 2 Dino Ferrari Centre, Department of Pathophysiology and Transplantation (DEPT), University of Milan, 20122 Milan, Italy; 3 Neurology Unit, IRCCS Fondazione Ca’ Granda Ospedale Maggiore Policlinico, Milan, Italy; 4 Department of Precision and Regenerative Medicine and Ionian Area - DiMePRe-J, Section of Pharmacology, University of Bari Aldo Moro, 70124 Bari, Italy; 5 Department of Precision and Regenerative Medicine and Ionian Area - DiMePRe-J, Guido Baccelli Unit of Internal Medicine, School of Medicine, University of Bari Aldo Moro, 70124 Bari, Italy

**Keywords:** Pompe disease, Desensitization, Enzyme replacement therapy, Avalglucosidase, Basophil activation test, Omalizumab, Drug allergy

## Abstract

**Background:**

Pompe disease is a rare, progressive lysosomal storage disorder caused by acid α-glucosidase deficiency, leading to glycogen accumulation, proximal muscle weakness, and respiratory decline. Enzyme replacement therapy (ERT) significantly improves survival and stabilizes motor function, but IgE-mediated hypersensitivity reactions (HSRs) can critically compromise treatment, posing a major clinical challenge. Desensitization protocols allow temporary tolerance to ERT, yet breakthrough reactions may occur, necessitating adjunctive strategies such as omalizumab.

**Results:**

We report a 40-year-old woman with late-onset Pompe disease who developed severe IgE-mediated HSRs to alglucosidase alfa after years of uneventful therapy. Basophil activation testing (BAT) and serum-specific IgE confirmed an IgE-mediated mechanism. A 15-step, 5-bag desensitization protocol allowed temporary tolerance, but breakthrough reactions required therapy interruption. Upon switching to avalglucosidase alfa, BAT demonstrated IgE cross-reactivity, and a new desensitization protocol was implemented. Initial infusions were complicated by recurrent HSRs. The addition of subcutaneous omalizumab (300 mg monthly), administered two days before ERT, enabled safe reintroduction. Moreover, therapy was resumed gradually, starting at 50% of the target dose and escalating stepwise to the full therapeutic dose, resulting in uninterrupted treatment. Follow-up showed stable neuromuscular and respiratory function, progressive decline in BAT reactivity, and improved quality of life.

**Conclusions:**

This case highlights the critical role of BAT in diagnosing and monitoring IgE-mediated HSRs, the efficacy of individualized desensitization protocols, and the utility of omalizumab as an adjunctive therapy in refractory cases. In rare diseases like Pompe, documenting such integrated allergological strategies provides practical guidance for maintaining access to life-prolonging therapy and offers a reproducible framework for managing complex allergic complications.

## Introduction

Pompe disease, or glycogen storage disease type II, is a rare autosomal recessive disorder caused by the deficiency of acid α-glucosidase, leading to lysosomal glycogen accumulation, progressive muscle weakness, and respiratory decline [1]. The clinical spectrum ranges from the severe infantile-onset form to late-onset Pompe disease (LOPD), typically characterized by proximal limb-girdle weakness and respiratory insufficiency [2]. The introduction of enzyme replacement therapy (ERT) with recombinant human GAA has revolutionized the management of Pompe disease, significantly prolonging survival and stabilizing motor function [3]. Nevertheless, ERT administration is frequently complicated by adverse reactions that may critically affect treatment adherence and outcomes. These reactions can be broadly categorized into infusion-related reactions (IRRs), usually non-IgE-mediated and driven by cytokine release or complement activation, and hypersensitivity reactions (HSRs), which can be IgE-dependent and potentially life-threatening [4]. While IRRs often present with fever, chills, flushing, or flu-like symptoms and may improve by reducing the infusion rate or adding standard premedication, IgE-mediated HSRs manifest as urticaria, angioedema, bronchospasm, or even anaphylaxis, and usually require more advanced allergological strategies.

Among these, desensitization protocols represent the mainstay of management in patients with recurrent or severe HSRs who cannot otherwise continue ERT. Rapid drug desensitization induces a temporary state of tolerance by administering the culprit drug in escalating doses through multiple steps and bags with increasing concentrations [5]. The underlying mechanism is not fully elucidated, but current evidence suggests that during desensitization, the progressive administration of subtherapeutic doses of the enzyme may occupy IgE molecules bound to FcεRI without inducing effective cross-linking, or, alternatively, may induce rapid internalization and downregulation of these receptors, thereby making mast cells and basophils temporarily unresponsive to further antigen exposure [6]. This controlled exposure enables patients to tolerate subsequent full therapeutic doses safely, though tolerance is transient and requires the protocol to be repeated at each infusion. Desensitization has therefore become an essential tool in ensuring that patients with lysosomal storage disorders, including Pompe disease, can continue to benefit from ERT despite the occurrence of IgE-mediated HSRs [7]. Yet, a proportion of patients experience breakthrough reactions even under optimized desensitization, underlining the need for adjunctive strategies such as omalizumab, an anti-IgE monoclonal antibody increasingly reported as a valuable rescue option in refractory cases [8]. However, evidence on the combined use of desensitization and omalizumab, along with the application of basophil activation testing as a diagnostic and monitoring tool in Pompe disease, remains limited. Here, we report a case illustrating the clinical utility of this integrated allergological approach.

## Case presentation

We describe the case of a 40-year-old Italian woman with genetically confirmed LOPD (compound heterozygous GAA mutations c.3213T > G and c.1944_195del), diagnosed at the age of 29 years following onset of progressive proximal weakness and mild respiratory insufficiency. At baseline, serum creatine kinase was 580 U/L (normal < 170 U/L). Pulmonary function tests showed a forced vital capacity (FVC) of 68% predicted upright and 61% supine, consistent with diaphragmatic weakness. Neuromuscular assessment revealed hip flexors MRC 3/5, hip extensors MRC 3+/5, and shoulder abductors MRC 4/5. Functional capacity was reduced, with a six-minute walking test (6MWT) distance of 385 m (62% predicted) and a Quick Motor Function Test (QMFT) score of 38/64. Notably, she had no history of atopy, drug allergy, or previous episodes of urticaria or angioedema.

Since 2013 she had been receiving alglucosidase alfa 20 mg/kg biweekly with stable motor function and no adverse events until May 2024, when she developed her first HSR consisting of generalized urticaria, angioedema, bronchospasm, and hypotension within 30 min of infusion, which typically lasted approximately 4 h. Subsequent attempts with premedication, including cetirizine and prednisone, did not prevent similar reactions, ultimately requiring epinephrine. An allergology work-up was carried out performing the Basophil Activation Test (BAT), which is a functional in vitro assay that measures the activation of basophils after exposure to the suspected allergen by detecting surface markers such as CD63, thus providing evidence of an IgE-mediated mechanism. The test was positive, with 45% CD63 expression (cut-off 15) and, together with the detection of serum-specific IgE to alglucosidase alfa (0.48 kU/L), supported the diagnosis of an IgE-mediated hypersensitivity reaction. Moreover, BAT provided functional evidence of basophil activation in response to the drug. Skin testing was not performed, as non-irritant concentrations for recombinant enzymes are not standardized and systemic risks were considered significant.

In September 2024, in light of the previous reactions and the need of reintroducing the ERT, a 5 bag, 15-step desensitization protocol over 10 h enabled the administration of the full therapeutic dose (20 mg/kg) under premedication with prednisone and cetirizine. Initially tolerated, this approach allowed several months of treatment, but in February 2025 a breakthrough systemic reaction recurred with urticaria, hypotension and desaturation, requiring therapy discontinuation and epinephrine.

**Fig. 1:**
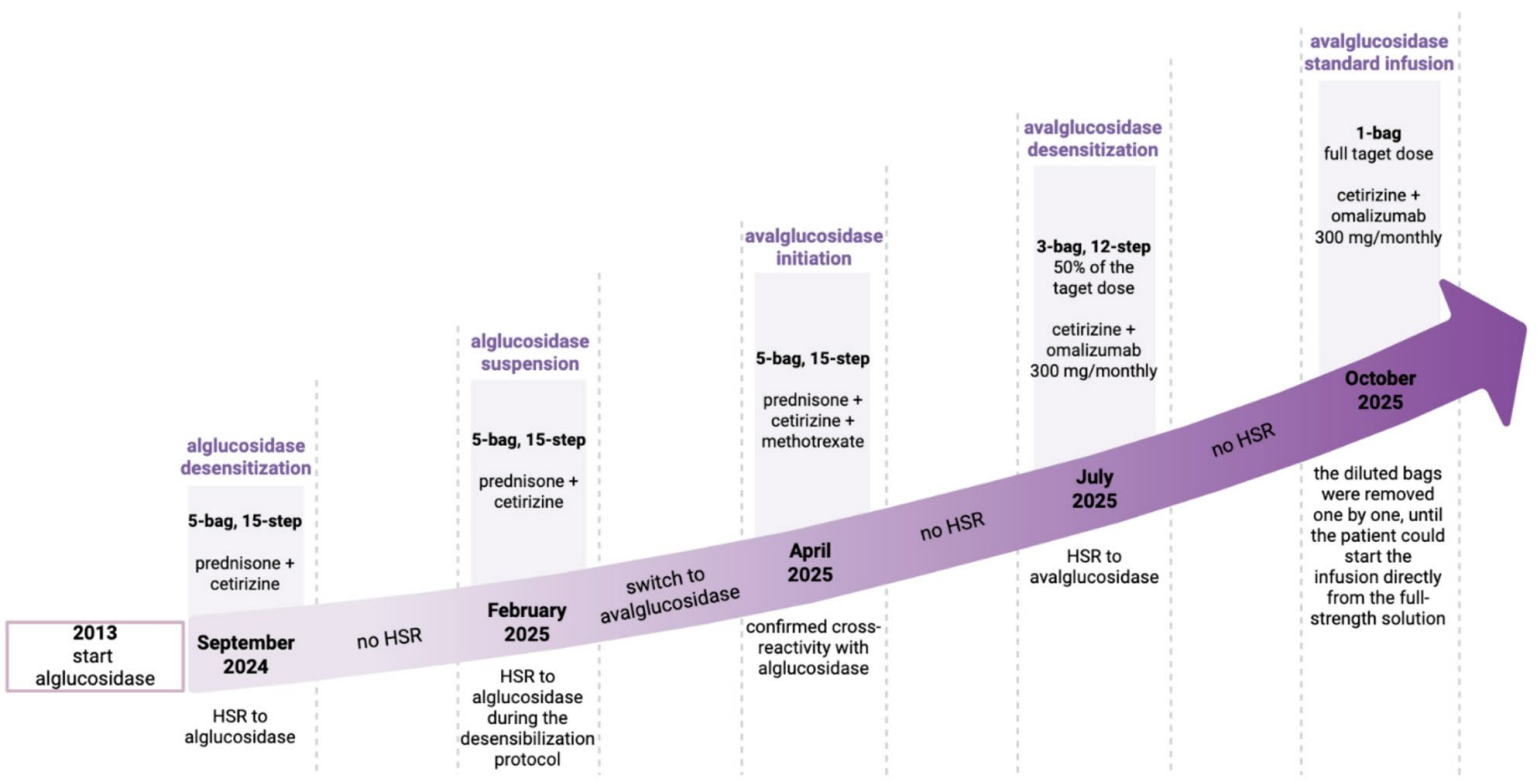
Chronological overview of clinical management

In April 2025, since avalglucosidase alfa had just become available as a new therapeutic option, the patient was switched to this drug at the 20 mg/kg dose: 900 mg every other week. Given the known structural similarity and potential cross-reactivity between alglucosidase alfa and avalglucosidase alfa, an allergological work-up was performed prior to infusion. The BAT confirmed IgE sensitization also to avalglucosidase alfa (42%), and therefore the desensitization protocol previously adopted for alglucosidase alfa was maintained, this time reinforced with low-dose methotrexate (10 mg/week) as additional premedication. Nevertheless, in June 2025 the patient experienced another systemic HSR, with pruritus, diffuse rash, and dyspnea within 30 min of the infusion, again requiring suspension. Given the persistence of severe IgE-mediated HSRs despite optimal management, subcutaneous omalizumab 300 mg every four weeks was introduced in July 2025, administration taking place two days before re-exposure. Then, avalglucosidase alfa was reintroduced using a 12-step, 3-bag desensitization protocol (Table 1) with progressive concentrations (1:100, 1:10, 1:1), initially tolerated at 10 mg/kg (target dose: 450 mg) and thus subsequently escalated to 15 mg/kg (target dose: 675 mg). Premedication consisted of cetirizine 10 mg. Once tolerance was achieved, the protocol was progressively simplified: one bag at a time was removed, starting from the most diluted, until the patient could begin infusions directly from the full-strength solution (Fig. 1).

**Table 1:** Desensitization protocol for avalglucosidase alfa

Step	Bag (dilution)	Infusion rate (ml/h)	Time (min)	Delivered dose (mg)
1	1/100	2.5	15	0.01125
2	1/100	5	15	0.0225
3	1/100	10	15	0.045
4	1/100	20	15	0.09
5	1/10	5	15	0.225
6	1/10	10	15	0.45
7	1/10	20	15	0.9
8	1/10	40	15	1.8
9	1/1	10	15	4.5
10	1/1	20	15	9
11	1/1	40	15	18
12	1/1	80	166.875	400.5
**Total**			**331.875**	**435.5**

Target dose: 450 mg

Bag 1/1: 450 mg of avalglucosidase alfa in 250 ml of 5% glucose solution [1.8 mg/ml];

Bag 1/10: 10 ml of 1/1 dilution in 90 ml of 5% glucose solution [0.18 mg/ml];

Bag 1/100: 10 ml of 1/10 dilution in 90 ml of 5% glucose solution [0.018 mg/dl]

At present, she receives/is receiving avalglucosidase alfa at the full standard dose of 20 mg/kg (900 mg) directly from the mother solution according to the leaflet schedule (standard protocol), with premedication limited to cetirizine alone, so far without any adverse events. Follow-up monitoring demonstrated a decline in BAT reactivity to 12%, suggesting an immunological modulation, and the patient reports significant improvement in quality of life, with uninterrupted ERT and no further hospitalizations. Neuromuscular follow-up showed stable disease: FVC increased to 74% upright, QMFT 41/64, and 6MWT 425 m, indicating preserved respiratory and motor function under continued therapy.

## Discussion

HSRs to ERT represent a challenging and still underrecognized complication in the long-term management of Pompe disease. Although the overall safety profile of alglucosidase and avalglucosidase is favorable, IgE-mediated reactions may occur even after years of uneventful infusions, abruptly threatening treatment continuity and patient outcomes. In such cases, allergological assessment and individualized therapeutic strategies become essential to ensure sustained access to ERT, which remains fundamental for prolonging survival and maintaining neuromuscular and respiratory function. Desensitization protocols have been increasingly applied in lysosomal storage disorders and currently represent the cornerstone strategy to maintain ERT in patients with IgE-mediated HSRs, although standardized approaches are still lacking.

This case illustrates several points of clinical relevance. IgE-mediated HSRs to ERT may occur even after years of uneventful treatment, and their severity can critically compromise therapy in otherwise stable patients. In our patient, both the BAT and the detection of drug-specific IgE confirmed the allergic nature of the reactions. Importantly, BAT was not only essential in diagnosis but also served as a valuable follow-up tool, demonstrating a progressive decline in basophil reactivity during treatment with omalizumab. In this context, BAT may represent a dynamic functional biomarker, reflecting the degree of IgE-mediated cellular responsiveness to the culprit drug. Compared with in vivo testing, BAT offers a safer in vitro alternative, particularly in patients at high risk of severe reactions. Similar applications of BAT in drug hypersensitivity reactions, including biologics and chemoterapy, have been reported, supporting its role as a complementary diagnostic and monitoring tool in complex allergic conditions [9, 10]. This represents an added strength, as BAT provides a safe, functional, and repeatable in vitro alternative in situations where in vivo testing is contraindicated.

Desensitization played a central role as a first-line allergological tool, allowing the reintroduction of ERT in greater safety by inducing a temporary state of tolerance through stepwise exposure to increasing concentrations of the enzyme. Although breakthrough reactions may occur despite optimized protocols, desensitization remains the cornerstone strategy for maintaining access to life-saving therapy.

In this context, the introduction of omalizumab proved decisive. Monthly administration of 300 mg was sufficient to suppress IgE-mediated reactivity and to enable the safe and sustained reintroduction of avalglucosidase alfa, with the remarkable finding that a single monthly injection provided effective protection for two biweekly infusions. Beyond its ability to neutralize circulating IgE, omalizumab, as it is well known, also progressively downregulates FcεRI expression on mast cells and basophils, thereby reducing their responsiveness over time. This dual mechanism not only explains the immediate control of allergic reactivity, but may also contribute to the long-term immunological modulation observed in our patient, as reflected by the decline in BAT reactivity.

Moreover, in our patient, beyond the combination of desensitization and omalizumab, an additional safety measure was the gradual escalation of drug dosage across consecutive infusions: tolerance was first established at 10 mg/kg, then consolidated at 15 mg/kg, and only thereafter was full treatment at 20 mg/kg resumed. This stepwise increase minimized the risk of severe reactions during the reintroduction phase.

Nevertheless, this report has limitations. Skin testing was not performed, as the patient had experienced severe anaphylaxis and the risk of provoking a further reaction was deemed excessive. Moreover, non-irritant concentrations for recombinant enzymes are not standardized, and ideally testing should begin with a skin prick test before proceeding to intradermal testing, which carries higher systemic risk. For these reasons, an in vitro approach was preferred.

Recent reports have also addressed desensitization in Pompe disease. Gendive et al. described a case of infantile-onset Pompe disease in which desensitization to both alglucosidase alfa and avalglucosidase alfa was successfully performed [11]. The protocol was rapid and effective, and intradermal skin testing was positive for both enzymes, supporting the allergic basis. However, drug-specific IgE were not assessed, leaving the serological characterization incomplete. Mendelsohn et al. reported a patient with late-onset Pompe disease who developed recurrent infusion reactions to both enzymes and was successfully managed with an individualized desensitization protocol combined with omalizumab [12]. In that case, intradermal skin testing was positive, and anti-drug specific IgE were detected at very low levels. This report provided valuable clinical confirmation that omalizumab can be integrated into desensitization regimens.

These observations confirm that alglucosidase alfa and avalglucosidase alfa are cross-reactive at the immunological level, which has practical implications for clinical management. In patients with IgE-mediated HSRs to alglucosidase alfa, it is therefore advisable to perform an allergological work-up - including skin tests, when feasible, and BAT as a complementary or alternative tool - before initiating avalglucosidase alfa [13, 14]. Such pre-infusion testing may help to stratify risk, anticipate cross-reactive hypersensitivity, and guide the safest therapeutic approach.

Taken together, the available literature, along with our case, support the concept that desensitization is the fundamental approach for managing IgE-mediated HSRs to ERT, while adjunctive omalizumab can secure tolerance in refractory cases, as described for other recombinant enzymes [15, 16].

Our case underscores the added value of the BAT as a both diagnostic and monitoring tool, supporting its role as a complement and, in selected high-risk cases, a potential alternative to skin testing, in situations where in vivo procedures are unsafe or not standardized. In addition, the desensitization strategy, reinforced by a cautious stepwise dose escalation across consecutive infusions and the use of omalizumab as an adjunctive safety measure, minimized the risk of severe hypersensitivity reactions. This integrated allergological approach ultimately enabled the safe continuation of full-dose ERT without recurrence of hypersensitivity, providing a practical and reproducible framework for managing similarly complex cases.

## Data Availability

Datasets are available on reasonable request from the corresponding author.

## Abbreviation

BAT: Basophil activation test
ERT: Enzyme replacement therapy
FVC: Forced vital capacity
HSR: Hypersensitivity reaction
LOPD: Late-onset Pompe disease
QMFT: Quick Motor Function Test
6MWT: Six-minute walking test
